# Supplementary material for: The Use of Crystal Violet Degradation Products for Ballpoint Pen Ink Manuscript Dating
Source: Molecules. 2023 Sep 4;28(17):6429. doi: 10.3390/molecules28176429 (PMC10490468; doi:10.3390/molecules28176429)
Supplement: Supplementary file 1 [file molecules-28-06429-s001.zip › molecules-2586922-supplementary.pdf]

Table S1. Blue Inoxcrom®: Person, Tau B and Rho Spearman correlations. Level of significance given in brackets.

Variables (Est y p-valor)		Phenol (PH)	NNDAB	NNAPH	Michler Ketone	Victoria Blue R	Ethyl Violet	Penta-PRS	Tetra-PRS	Tri-PRS	Di-PRS
Time (Month)	Pearson	-.473* (.023)	-.526** (.010)	-.678** (.000)	-.558** (.006)	-.869** (.000)	.119^{NS} (.589)	.390^{NS} (.066)	.019^{NS} (.931)	.607** (.002)	-.316^{NS} (.142)
	Tau B	-.500** (.001)	-.692** (.000)	-.478** (.001)	-.415** (.006)	-.796** (.000)	.013^{NS} (.935)	.344* (.022)	-.241^{NS} (.107)	.464** (.004)	-.344* (.022)
	Rho Spearman	-.650** (.001)	-.838** (.000)	-.723** (.000)	-.549** (.007)	-.920** (.000)	.013^{NS} (.953)	.448* (.032)	-.320^{NS} (.136)	.625** (.001)	-.400^{NS} (.058)
	Covariance	-.153	-.050	-.418	-.127	-.874	.086	16.057	.073	2.164	-.655
Phenol (PH)	Pearson		.918** (.000)	.355^{NS} (.096)	.108^{NS} (.624)	.364^{NS} (.087)	-.068^{NS} (.756)	-.189^{NS} (.387)	-.042^{NS} (.848)	-.375^{NS} (.078)	.093^{NS} (.673)
	Tau B		.515** (.002)	.436** (.004)	.268^{NS} (.076)	.377* (.013)	.144^{NS} (.357)	-.004^{NS} (.979)	.212^{NS} (.160)	-.242^{NS} (.136)	.196^{NS} (.194)
	Rho Spearman		.620** (.002)	.577** (.004)	.334^{NS} (.120)	.481* (.020)	.163^{NS} (.456)	-.033^{NS} (.881)	.307^{NS} (.155)	-.329^{NS} (.125)	.247^{NS} (.256)
	Covariance		.000	.000	.000	.000	.000	-.002	.000	.000	.000
NNDAB	Pearson			.411^{NS} (.051)	.284^{NS} (.189)	.466* (.025)	-.148^{NS} (.502)	-.304^{NS} (.158)	-.025^{NS} (.910)	-.488* (.018)	.223^{NS} (.307)
	Tau B			.425** (.010)	.356* (.030)	.620** (.000)	-.037^{NS} (.829)	-.454** (.006)	.178^{NS} (.278)	-.569** (.001)	.316^{NS} (.054)
	Rho Spearman			.544** (.007)	.467* (.025)	.791** (.000)	-.045^{NS} (.838)	-.569** (.005)	.277^{NS} (.201)	-.689** (.000)	.439* (.036)
	Covariance			.000	.000	.000	.000	-.001	.000	.000	.000
NNAPH	Pearson				.482* (.020)	.644** (.001)	.040^{NS} (.857)	-.249^{NS} (.251)	.020^{NS} (.929)	-.475* (.022)	.241^{NS} (.269)
	Tau B				.447** (.003)	.452** (.003)	.155^{NS} (.317)	-.154^{NS} (.303)	.194^{NS} (.196)	-.326* (.042)	.265^{NS} (.077)
	Rho Spearman				.574** (.004)	.636** (.001)	.200^{NS} (.359)	-.224^{NS} (.304)	.263^{NS} (.226)	-.464* (.026)	.330^{NS} (.124)
	Covariance				.000	.000	.000	-.006	.000	-.001	.000
Michler Ketone	Pearson					.565** (.005)	-.238^{NS} (.274)	.038^{NS} (.862)	.602** (.002)	-.302^{NS} (.162)	.885** (.000)
	Tau B					.532** (.000)	.029^{NS} (.850)	-.154^{NS} (.303)	.636** (.000)	-.198^{NS} (.218)	.723** (.000)
	Rho Spearman					.718** (.000)	.017^{NS} (.940)	-.300^{NS} (.164)	.829** (.000)	-.315^{NS} (.143)	.899** (.000)
	Covariance					.000	.000	.000	.000	.000	.000
Victoria Blue R	Pearson						-.179^{NS} (.415)	-.435* (.038)	-.023^{NS} (.916)	-.521* (.011)	.335^{NS} (.118)
	Tau B						-.064^{NS} (.684)	-.428** (.005)	.364* (.016)	-.423** (.009)	.492** (.001)
	Rho Spearman						-.097^{NS} (.658)	-.503* (.014)	.501* (.015)	-.568** (.005)	.611** (.002)
	Covariance						.000	-.017	.000	-.002	.001
Ethyl Violet	Pearson							.390^{NS} (.066)	.081^{NS} (.714)	.347^{NS} (.105)	-.247^{NS} (.256)
	Tau B							.306* (.048)	.046^{NS} (.766)	.205^{NS} (.219)	-.013^{NS} (.935)
	Rho Spearman							.391^{NS} (.065)	.057^{NS} (.796)	.225^{NS} (.302)	-.080^{NS} (.716)
	Covariance							.011	.000	.001	.000
Penta-PRS	Pearson								.774** (.000)	.801** (.000)	.292^{NS} (.176)
	Tau B								.004^{NS} (.979)	.639** (.000)	-.178^{NS} (.235)
	Rho Spearman								.046^{NS} (.833)	.786** (.000)	-.243^{NS} (.264)
	Covariance								.116	.111	.024
Tetra-PRS	Pearson									.397^{NS} (.061)	.830** (.000)
	Tau B									.032^{NS} (.841)	.818** (.000)
	Rho Spearman									.056^{NS} (.800)	.919** (.000)
	Covariance									.005	.006
Tri-PRS	Pearson										-.095^{NS} (.666)
	Tau B										-.097^{NS} (.548)
	Rho Spearman										-.198^{NS} (.366)
	Covariance										-.001

^{NS} = NO significant * =Significant ** = Highly significant.

Table S2. Black Inoxcrom®: Person, Tau B and Rho Spearman correlations. Level of significance given in brackets.

Variables (Est y p-valor)		Phenol (PH)	NNDAB	NNAPH	Metanil Yellow	Ethyl Violet	Penta-PRS	Tetra-PRS	Tri-PRS	Di-PRS
**Time (Month)**	**Pearson**	**-.610** (.001)**	.281^{NS} (.174)	**-.523** (.007)**	-.325^{NS} (.113)	-.086^{NS} (.683)	**-.932** (.000)**	**-.818** (.000)**	-.133^{NS} (.526)	**-.646** (.000)**
	**Tau B**	**-.727** (.000)**	.293^{NS} (.063)	-.140^{NS} (.327)	-.073^{NS} (.607)	-.116^{NS} (.454)	**-.720** (.000)**	**-.580** (.000)**	-.117^{NS} (.413)	**-.507** (.000)**
	**Rho Spearman**	**-.878** (.000)**	**.405* (.045)**	-.314^{NS} (.127)	-.229^{NS} (.270)	-.118^{NS} (.574)	**-.865** (.000)**	**-.748** (.000)**	-.120^{NS} (.567)	**-.672** (.000)**
	**Covariance**	-.112	.003	-.095	-2.354	-.022	-37.039	-1.993	-.212	-.904
**Phenol (PH)**	**Pearson**		.007^{NS} (.972)	.358^{NS} (.079)	-.050^{NS} (.812)	-.127^{NS} (.547)	**.535** (.006)**	**.639** (.001)**	-.174^{NS} (.404)	**.619** (.001)**
	**Rho Spearman**		-.198^{NS} (.209)	.227^{NS} (.112)	.120^{NS} (.400)	.004^{NS} (.979)	**.607** (.000)**	**.653** (.000)**	.204^{NS} (.154)	**.593** (.000)**
	**Tau B**		-.279^{NS} (.176)	**.396* (.05)**	.225^{NS} (.279)	-.036^{NS} (.866)	**.818** (.000)**	**.842** (.000)**	.226^{NS} (.278)	**.777** (.000)**
	**Covariance**		.000	.000	.000	.000	.004	.000	.000	.000
**NNDAB**	**Pearson**			-.254^{NS} (.220)	**-.461* (.020)**	.236^{NS} (.256)	-.352^{NS} (.084)	**-.406* (.044)**	-.154^{NS} (.463)	-.389^{NS} (.055)
	**Rho Spearman**			-.224^{NS} (.155)	**-.422** (.007)**	.093^{NS} (.588)	**-.318* (.043)**	**-.448** (.004)**	-.208^{NS} (.189)	**-.422** (.007)**
	**Tau B**			-.280^{NS} (.176)	**-.552** (.004)**	.122^{NS} (.561)	**-.405* (.045)**	**-.551** (.004)**	-.245^{NS} (.237)	**-.535** (.006)**
	**Covariance**			.000	.000	.000	.000	.000	.000	.000
**NNAPH**	**Pearson**				**.582** (.002)**	-.243^{NS} (.241)	**.533** (.006)**	**.559** (.004)**	.206^{NS} (.323)	**.535** (.006)**
	**Rho Spearman**				**.387** (.007)**	-.100^{NS} (.519)	.127^{NS} (.375)	**.347* (.015)**	.191^{NS} (.183)	**.340* (.017)**
	**Tau B**				**.577** (.003)**	-.144^{NS} (.491)	.305^{NS} (.139)	**.486* (.014)**	.259^{NS} (.210)	**.472* (.017)**
	**Covariance**				.001	.000	.004	.000	.000	.000
**Metanil Yellow**	**Pearson**					-.240^{NS} (.248)	**.497* (.011)**	**.490* (.013)**	.329^{NS} (.108)	**.440* (.028)**
	**Rho Spearman**					-.124^{NS} (.423)	.273^{NS} (.055)	**.387** (.007)**	**.332* (.021)**	**.353* (.013)**
	**Tau B**					-.196^{NS} (.349)	**.412* (.041)**	**.571** (.003)**	**.486* (.014)**	**.544** (.005)**
	**Covariance**					.000	.138	.008	.004	.004
**Ethyl Violet**	**Pearson**						.095^{NS} (.650)	-.297^{NS} (.149)	-.157^{NS} (.455)	**-.496* (.012)**
	**Rho Spearman**						.140^{NS} (.366)	-.156^{NS} (.314)	-.092^{NS} (.552)	-.260^{NS} (.093)
	**Tau B**						.169^{NS} (.421)	-.315^{NS} (.126)	-.191^{NS} (.361)	**-.432* (.031)**
	**Covariance**						.001	.000	.000	.000
**Penta-PRS**	**Pearson**							**.854** (.000)**	.313^{NS} (.128)	**.654** (.000)**
	**Rho Spearman**							**.580** (.000)**	**.318* (.026)**	**.493** (.001)**
	**Tau B**							**.781** (.000)**	**.479* (.015)**	**.688** (.000)**
	**Covariance**							.080	.019	.035
**Tetra-PRS**	**Pearson**								.318^{NS} (.121)	**.948** (.000)**
	**Rho Spearman**								**.439** (.002)**	**.887** (.000)**
	**Tau B**								**.430* (.032)**	**.970** (.000)**
	**Covariance**								.001	.003
**Tri-PRS**	**Pearson**									.270^{NS} (.191)
	**Rho Spearman**									**.486** (.001)**
	**Tau B**									**.431* (.031)**
	**Covariance**									.001

^{NS} = NO significant * =Significant ** = Highly significant.

Table S3. Blue Sigma®: Person, Tau B and Rho Spearman correlations. Level of significance given in brackets

Variables *(Est y p-valor)*		Phenol (PH)	NNDAB	NNAPH	Michler Ketone	Ethyl Violet	Penta-PRS	Tetra-PRS	Tri-PRS	Di-PRS
Time (Month)	Pearson	-.512** (.006)	-.505** (.007)	-.467* (.014)	-.839** (.000)	-.614** (.001)	-.340^{NS} (.083)	-.557** (.003)	-.679** (.000)	.406* (.036)
	Tau B	-.487** (.000)	-.468** (.001)	-.123^{NS} (.370)	-.527** (.000)	-.385** (.005)	-.208^{NS} (.128)	-.242^{NS} (.076)	-.419** (.002)	.397** (.006)
	Rho Spearman	-.629** (.000)	-.625** (.000)	-.283^{NS} (.153)	-.675** (.000)	-.542** (.004)	-.301^{NS} (.127)	-.415* (.031)	-.585** (.001)	.595** (.001)
	Covariance	-.118	-.063	-.106	-.968	-.477	-2.202	-13.971	-4.128	1.476
Phenol (PH)	Pearson		.314^{NS} (.111)	.283^{NS} (.153)	.478* (.012)	.338^{NS} (.085)	.120^{NS} (.550)	.090^{NS} (.656)	.428* (.026)	-.212^{NS} (.289)
	Tau B		.143^{NS} (.297)	.225^{NS} (.100)	.402** (.003)	.328* (.017)	.231^{NS} (.091)	.117^{NS} (.393)	.373** (.006)	-.230^{NS} (.109)
	Rho Spearman		.240^{NS} (.228)	.391* (.044)	.567** (.002)	.486* (.01)	.301^{NS} (.127)	.204^{NS} (.308)	.545** (.003)	-.308^{NS} (.117)
	Covariance		.000	.000	.000	.000	.000	.001	.001	.000
NNDAB	Pearson			-.204^{NS} (.308)	.108^{NS} (.591)	-.067^{NS} (.742)	-.258^{NS} (.194)	.132^{NS} (.513)	-.023^{NS} (.910)	-.506** (.007)
	Tau B			-.200^{NS} (.144)	.057^{NS} (.677)	-.074^{NS} (.588)	-.160^{NS} (.243)	.001^{NS} (.999)	-.040^{NS} (.770)	-.463** (.001)
	Rho Spearman			-.204^{NS} (.307)	.093^{NS} (.643)	-.067^{NS} (.742)	-.204^{NS} (.308)	.029^{NS} (.887)	-.026^{NS} (.899)	-.602** (.001)
	Covariance			.000	.000	.000	.000	.000	.000	.000
NNAPH	Pearson				.698** (.000)	.726** (.000)	.701** (.000)	.605** (.001)	.708** (.000)	.188^{NS} (.347)
	Tau B				.504** (.000)	.578** (.000)	.584** (.000)	.436** (.001)	.556** (.000)	.074^{NS} (.604)
	Rho Spearman				.677** (.000)	.723** (.000)	.779** (.000)	.586** (.001)	.713** (.000)	.097^{NS} (.631)
	Covariance				.000	.000	.001	.004	.001	.000
Michler Ketone	Pearson					.886** (.000)	.725** (.000)	.580** (.002)	.947** (.000)	-.158^{NS} (.431)
	Tau B					.789** (.000)	.635** (.000)	.385** (.005)	.858** (.000)	-.186^{NS} (.194)
	Rho Spearman					.941** (.000)	.837** (.000)	.518** (.006)	.962** (.000)	-.299^{NS} (.130)
	Covariance					.001	.006	.018	.007	-.001
Metanil Yellow	Pearson					.867** (.000)	.801** (.000)	.524** (.005)	.920** (.000)	-.058^{NS} (.774)
	Tau B					.766** (.000)	.738** (.000)	.464** (.001)	.755** (.000)	-.105^{NS} (.462)
	Rho Spearman					.935** (.000)	.911** (.000)	.611** (.001)	.929** (.000)	-.129^{NS} (.522)
	Covariance					.001	.010	.026	.011	.000
Penta-PRS	Pearson							.693** (.000)	.842** (.000)	.405* (.036)
	Tau B							.487** (.000)	.721** (.000)	.099^{NS} (.489)
	Rho Spearman							.659** (.000)	.877** (.000)	.101^{NS} (.618)
	Covariance							.124	.037	.011
Tetra-PRS	Pearson								.626** (.000)	.368^{NS} (.059)
	Tau B								.470** (.001)	.180^{NS} (.209)
	Rho Spearman								.609** (.001)	.271^{NS} (.171)
	Covariance								.105	.037
Tri-PRS	Pearson									.074^{NS} (.715)
	Tau B									-.043^{NS} (.762)
	Rho Spearman									-.102^{NS} (.611)
	Covariance									.002

^{NS} = NO significant * =Significant ** = Highly significant.

Table S4. Black Sigma®: Person, Tau B and Rho Spearman correlations. Level of significance given in brackets

		Phenol (PH)	NNDAB	NNAPH	Michler Ketone	Victoria Blue R	Penta-PRS	Tetra-PRS	Tri-PRS	Di-PRS	Mono-PRS
Time (Month)	Pearson	-.342^{NS} (.07)	-.609** (.000)	-.525** (.003)	-.941** (.000)	-.968** (.000)	-.848** (.000)	-.757** (.000)	-.382* (.041)	-.586** (.001)	-.378* (.043)
	Tau B	-.739** (.000)	-.542** (.000)	-.246^{NS} (.061)	-.704** (.000)	-.872** (.000)	-.798** (.000)	-.631** (.000)	-.236 (.072)	-.458** (.000)	-.236^{NS} (.072)
	Rho	-.908** (.000)	-.717** (.000)	-.407* (.028)	-.836** (.000)	-.964** (.000)	-.915** (.000)	-.818** (.000)	-.384* (.040)	-.630** (.000)	-.384* (.040)
	Cov.	-.180	-.040	-.074	-.563	-1.113	-11.871	-1.564	-.428	-.827	-.426
Phenol (PH)	Pearson		.531** (.003)	.157^{NS} (.417)	.232^{NS} (.226)	.386* (.039)	.444* (.016)	.270^{NS} (.157)	.052^{NS} (.790)	.157^{NS} (.416)	.050^{NS} (.796)
	Tau B		.448** (.001)	.310* (.018)	.621** (.000)	.759** (.000)	.773** (.000)	.724** (.000)	.379** (.004)	.552** (.000)	.379** (.004)
	Rho		.632** (.000)	.478** (.009)	.826** (.000)	.926** (.000)	.933** (.000)	.877** (.000)	.557** (.002)	.730** (.000)	.557** (.002)
	Cov.		.000	.000	.000	.000	.003	.000	.000	.000	.000
NNDAB	Pearson			.065^{NS} (.737)	.394* (.035)	.599** (.001)	.791** (.000)	.535** (.003)	.168^{NS} (.384)	.348^{NS} (.064)	.175^{NS} (.363)
	Tau B			.034^{NS} (.793)	.335* (.011)	.493** (.000)	.507** (.000)	.409** (.002)	.281* (.032)	.345** (.009)	.281* (.032)
	Rho			.028^{NS} (.885)	.530** (.003)	.688** (.000)	.690** (.000)	.598** (.001)	.362^{NS} (.054)	.467* (.011)	.362^{NS} (.054)
	Cov.			.000	.000	.000	.001	.000	.000	.000	.000
NNAPH	Pearson				.651** (.000)	.597** (.001)	.355^{NS} (.059)	.347^{NS} (.065)	.159^{NS} (.411)	.271^{NS} (.155)	.150^{NS} (.436)
	Tau B				.315* (.016)	.345** (.009)	.261* (.047)	.310* (.018)	.182^{NS} (.165)	.335* (.011)	.182^{NS} (.165)
	Rho				.477** (.009)	.501** (.006)	.411* (.027)	.456* (.013)	.218^{NS} (.257)	.417* (.024)	.218^{NS} (.257)
	Cov.				.000	.000	.001	.000	.000	.000	.000
Michler Ketone	Pearson					.940** (.000)	.711** (.000)	.755** (.000)	.484** (.008)	.648** (.000)	.478** (.009)
	Tau B					.773** (.000)	.581** (.000)	.709** (.000)	.483** (.000)	.626** (.000)	.483** (.000)
	Rho					.892** (.000)	.765** (.000)	.879** (.000)	.691** (.000)	.830** (.000)	.691** (.000)
	Cov.					.001	.006	.001	.000	.001	.000
Victoria Blue R	Pearson						.881** (.000)	.798** (.000)	.423* (.022)	.627** (.000)	.420* (.023)
	Tau B						.778** (.000)	.729** (.000)	.335* (.011)	.557** (.000)	.335* (.011)
	Rho						.928** (.000)	.893** (.000)	.530** (.003)	.743** (.000)	.530** (.003)
	Cov.						.014	.002	.001	.001	.001
Penta-PRS	Pearson							.812** (.000)	.407* (.028)	.600** (.001)	.404* (.030)
	Tau B							.685** (.000)	.320* (.015)	.483** (.000)	.320* (.015)
	Rho							.859** (.000)	.476** (.009)	.670** (.000)	.476** (.009)
	Cov.							.024	.006	.012	.006
Tetra-PRS	Pearson								.854** (.000)	.951** (.000)	.852** (.000)
	Tau B								.586** (.000)	.788** (.000)	.586** (.000)
	Rho								.773** (.000)	.926** (.000)	.773** (.000)
	Cov.								.002	.003	.002
Tri-PRS	Pearson									.966** (.000)	.999** (.000)
	Tau B									.778** (.000)	.999** (.000)
	Rho									.916** (.000)	.999** (.000)
	Cov.									.002	.001
Di-PRS	Pearson										.967** (.000)
	Tau B										.778** (.000)
	Rho										.916** (.000)
	Cov.										.002

^{NS} = NO significant * =Significant ** = Highly significant.
